# Comparison of Two Different Doses of Nalbuphine With Isobaric Ropivacaine in Patients Undergoing Lower Segment Cesarean Section Under Subarachnoid Block: A Randomized Controlled Trial

**DOI:** 10.7759/cureus.40558

**Published:** 2023-06-17

**Authors:** Shivam Shekhar, Rajesh S Rautela, Sujata Chaudhary, Sony Sony

**Affiliations:** 1 Anesthesiology, All India Institute of Medical Sciences, Rishikesh, Rishikesh, IND; 2 Anesthesiology, University College of Medical Sciences and Guru Teg Bahadur Hospital, Delhi, IND; 3 Anesthesiology, Vardhman Mahavir Medical College and Safdarjung Hospital, New Delhi, IND

**Keywords:** intrathecal opioids, adjuvants to subarachnoid block, post-operative analgesia, lower segment cesarean section, nalbuphine, isobaric ropivacaine, regional anesthesia

## Abstract

Introduction: Obstetric analgesia and anesthesia is a challenge in itself. It requires an understanding of the physiological changes during pregnancy and labor and the effect of anesthetic agents on the fetus and newborn. Because neuraxial techniques provide superior analgesia and materno-fetal benefits, their use have increased significantly over the past three decades or so. A combination of local anesthetics like ropivacaine with opioids like nalbuphine has been shown to have additive beneficial effects in subarachnoid block (SAB) in lower segment cesarean section (LSCS). However, the optimal dose combination of ropivacaine and nalbuphine to maximize their benefits and minimize side effects remains to be established. Our study has compared the clinical efficacy and safety of 0.75% isobaric ropivacaine (15 mg) with two different doses of nalbuphine (0.4 mg and 0.6 mg) when given intrathecally for LSCS in terms of quality of sensory and motor blocks, hemodynamic parameters, duration of effective analgesia, Apgar score in newborn, and associated side effects.

Method and materials: In this prospective, randomized, double-blind study, a total of 69 parturients between the age of 20-45 years, belonging to American Society of Anesthesiologists (ASA) grade I and II, undergoing cesarean section under SAB were evaluated. Patients were randomly allocated into three groups of 23 each by using the draw-of-lots technique. The patient and the observer were kept blinded as to which dose of drug (intrathecal) was being given to the patient. Patients in Group A received 0.75% isobaric ropivacaine 15 mg (2 ml) + 0.3 ml normal saline; patients in Group B received 0.75% isobaric ropivacaine 15 mg (2 ml) + 0.4 mg of nalbuphine (0.2 ml) + 0.1 ml normal saline; patients in Group C received 0.75% isobaric ropivacaine 15 mg (2 ml) + 0.6 mg of nalbuphine (0.3 ml). The total volume of drug solution in all three groups was 2.3 ml.

Result: We found that the time to onset of sensory block was shortest in Group A (5.87±1.290 minutes) followed by Group C (6.00±1.087 minutes) and Group B (6.17±1.696 minutes); time to two-segment regression of sensory block was longest in Group C (101.74±8.996 minutes) followed by Group B (85.87±15.348 minutes) and Group A (65.00±7.071 minutes); duration of effective analgesia was longest in Group C (206.09±18.766 minutes) followed by Group B (183.91±15.880 minutes) and Group A (121.74±11.833 minutes); and time from SAB to complete regression of motor block was longest in Group C (216.52±15.553 minutes) followed by Group B (203.48±20.138 minutes) and Group A (174.78±14.731 minutes). Side effects were comparable among all three groups.

Conclusion: The optimal dose combination in SAB for cesarean section was 15 mg of 0.75% isobaric ropivacaine + 0.6 mg nalbuphine, with minimal side effects.

## Introduction

Regional anesthesia has gained significant popularity among obstetric anesthesiologists in recent times. Spinal anesthesia is preferred over general anesthesia in cesarean section as the former is associated with better outcomes for both mothers and babies. Subarachnoid block (SAB) is safe, easy to perform, effective, inexpensive, and avoids complications of general anesthesia in parturients. It inhibits the stress response to surgery and has other advantages in the form of early ambulation, maternal satisfaction, and a lower rate of thromboembolism [1-3]. Although SAB is favored in cesarean section, it has some major drawbacks like short duration of action and limited postoperative analgesia when performed using only local anesthetics. It is important to use an anesthetic technique, which provides a longer duration of analgesia with minimal side effects and a low risk of toxicity to mother and baby.

Ropivacaine is an amide local anesthetic. It has a greater degree of sensory-motor differentiation. Ropivacaine's reduced lipophilicity compared to bupivacaine is associated with decreased potential for central nervous system and cardiac toxicity [4]. Various studies have been done to evaluate its intrathecal dosing based on baricity in both pregnant and non-pregnant patients. Ateser et al. conducted a study to evaluate the optimum dose of intrathecally-administered isobaric ropivacaine in pregnant women undergoing cesarean delivery [5]. They concluded that ropivacaine produces rapid induction along with a satisfactory anesthesia level, and that ropivacaine 15 mg and 20 mg dosing regimen are satisfactory for SAB.

Opioid adjuvants like morphine, fentanyl, sufentanil, and nalbuphine, used in SAB, have shown their beneficial effects in obstetric anesthesia with improved quality and duration of block and prolonged postoperative analgesia. However, intrathecal opioid also has side effects like nausea, vomiting, pruritus, sedation, respiratory depression, and urinary retention [6-9]. Nalbuphine is an opioid agonist-antagonist that is structurally related to oxymorphine and naloxone. It acts as an antagonist at the mu-receptor and as an agonist at the kappa-receptor. The onset of effect is rapid (5-10 minutes) and its duration of action is long (3-6 hours) because of a long plasma elimination t1/2 of five hours [10]. The analgesic efficacy of nalbuphine is comparable to morphine but nalbuphine has a better safety profile than morphine in the aspect of pruritus and respiratory depression [11]. Goma et al. conducted a study to compare intrathecal nalbuphine against intrathecal fentanyl as an adjuvant to bupivacaine for postoperative analgesia in patients undergoing cesarean delivery [12]. They found that both nalbuphine and fentanyl improved intraoperative analgesia, and prolonged early postoperative analgesia in lower segment cesarean section (LSCS).

Literature search did not reveal any study of isobaric ropivacaine with nalbuphine in LSCS under SAB. With this background, the present study was done to compare the efficacy of two different doses of nalbuphine with isobaric ropivacaine in patients undergoing LSCS under SAB. Our primary objective was to determine analgesic efficacy in terms of duration of effective analgesia (defined as the time from SAB to the patient’s first complaint of pain (visual analog scale (VAS) ≥3)) [13]. As our secondary objectives, we observed the quality of sensory and motor  block, Apgar score at one and five minutes, and side effects like nausea, hypotension, bradycardia, and pruritus [14].

## Materials and methods

Study design and subjects

This prospective, randomized controlled study was conducted at a tertiary care center, University College of Medical Sciences and Guru Teg Bahadur Hospital, New Delhi, India. Ethical clearance for this study was obtained from the Institutional Ethics Committee (Human Research), Board of University College of Medical Sciences, University of Delhi, New Delhi, India (approval number: 528/Anaes/7). Written informed consent was obtained from full-term parturients aged 20-45 years, height 140-180 cm, and planned for LSCS, and included in this study. Exclusion criteria were: patients having infection at injection site, coagulopathy, cardiac diseases, diabetes mellitus, pregnancy-induced hypertension, antepartum hemorrhage, fetal distress, seizure disorders, pre-existing neurological deficit, any spine deformity, any other contraindications of SAB, and any allergy to the drugs used in the study. The included patients were randomly allocated to one of the three groups (groups A, B, and C) using the draw-of-lots technique.

Pre-, intra-, and postoperative methodology

Drug combinations used in each group were: Group A: 15 mg of 0.75% isobaric ropivacaine (2 ml)+ normal saline 0.3 ml; Group B: 15 mg of 0.75% isobaric ropivacaine 2 ml+ 0.4 mg of nalbuphine (0.2 ml)+ normal saline 0.1 ml; Group C: 15 mg of 0.75% isobaric ropivacaine (2 ml)+ 0.6 mg of nalbuphine (0.3 ml).

The study drug was injected by the anesthesiologist, who was not involved in the observation or collection of data, in a double-blind fashion. Both the patient and the anesthesiologist were blinded to the patient's group assignment. Another anesthesiologist who was blinded to the randomization schedule performed all the recordings. A routine pre-anesthetic check-up of all the patients was done. During the pre-anesthetic check-up, VAS was explained to the patients for pain assessment. Adequate fasting of at least eight hours was confirmed before induction of anesthesia. Pre-medications, injection ranitidine 50 mg IV and injection metoclopramide 10 mg IV, were given half an hour before the surgery. After shifting the patients to the operation table, they were made to lie supine with a left lateral tilt of 15 degrees using a wedge. Essential monitors were attached. Baseline readings of heart rate (HR), blood pressure (BP), peripheral oxygen saturation (SpO2), and respiratory rate (RR) were noted. Co-loading was done with ringer’s lactate solution at the rate of 15 ml/kg of body weight during the procedure of giving SAB. Under all aseptic and antiseptic precautions, SAB was performed with 2.3 ml of the study drug injected in L3-L4 intervertebral space, using a 25-gauge quincke spinal needle, in the sitting position.

The level of sensory block was assessed by pinprick method in the mid-clavicular line bilaterally using a 26-gauge hypodermic needle every two minutes until the level was stabilized for three consecutive tests and the highest level of sensory block was noted. The quality of sensory block was assessed in terms of the time of onset of sensory block, maximum height of block, degree of loss of sensation, and two-segment regression of sensory block. The degree of loss of sensation was categorized as 1 = complete absence of sensation (excellent analgesia), 2 = sensation of motion only (good analgesia), 3 = mild discomfort but patient declining offer for additional analgesia (fair analgesia), 4 = intense discomfort with patient expressing a wish for additional analgesia (poor analgesia). The quality of motor block was assessed in terms of the Bromage scale [15]. The surgeon assessed the quality of muscular relaxation as “good”, “fair”, or “poor”.

Any episode of hypotension (defined as a fall in systolic blood pressure by more than 20% from the preoperative baseline value or systolic blood pressure by less than 90 mmHg) was noted and managed by exaggerating the left lateral tilt, increasing the rate of IV fluid and incremental IV boluses of mephentermine 3 mg. Intraoperative nausea, vomiting, shivering, headache, dizziness, bradycardia, pruritus, respiratory depression, and any other complications were noted and treated accordingly. The Apgar score of the newborn was noted at one minute and five minutes after birth. Cord blood collection was done immediately after delivery. The sample was analyzed by Combisys II arterial blood gas (ABG) machine (Eschweiler GmbH & Co. KG, Kiel, Germany) within 10 minutes of collection of the sample to assess the metabolic status of the neonate and rule out any perinatal asphyxia. A pH of less than 7 was considered perinatal asphyxia.

Postoperative routine monitoring was continued and time to two-segment regression of sensory block from maximum height of block was noted. HR, BP, SpO2, RR, sedation score, Bromage score, and VAS were noted every 15 minutes till 120 minutes of SAB and thereafter half hourly till 240 minutes from SAB. At the time of the patient's first complaint of pain (VAS≥3), IV paracetamol 1 gm was given as rescue analgesia, and time was noted; it was then continued eight-hourly for the next 24 hours. Duration of effective analgesia with SAB was defined as the duration of time from giving SAB to the patient’s first complaint of pain (VAS≥3). Side effects like nausea, vomiting, headache, dizziness, respiratory depression, pruritus, and urinary retention were noted as mentioned above and were treated accordingly. Sedation scoring was done using the five-point University of Michigan Sedation scale [16].

Sample size

In a previous study done by Khaw et al., the duration of effective analgesia in ropivacaine 15 mg was 128±40 minutes [17]. Expecting a minimum of 15% change in the duration of analgesia in the ropivacaine 15 mg + nalbuphine 0.4 mg group (Group B) and a minimum of 30% change in the duration of analgesia in the ropivacaine 15 mg + nalbuphine 0.6 mg group (Group C), with 80% power and 5% level of significance, we got a sample size of 69 subjects.

Statistical analysis

Repeated measure ANOVA was done to compare the results in all three groups followed by Tukey's test. A p-value of less than 0.05 was considered significant. One-way ANOVA followed by Tukey’s test was done to compare single-point quantitative data. Nominal data were compared with the help of Chi-square/Fisher's exact test. Apgar score, VAS, and degree of loss of sensation were compared using the Kruskal-Wallis test followed by the Mann-Whitney U test. The F-test was done to determine the feature importance score of various parameters. Linear regression and decision trees were used to validate the observation of the F-test. PASW Statistics for Windows, Version 18.0 (Released 2099; SPSS Inc., Chicago, United States) and R 3.2.2 (Released 2015; R Foundation for Statistical Computing, Vienna, Austria) were used for the analysis of the data, and Microsoft Word and Excel (Microsoft Corporation, Redmond, Washington, United States) were used to generate tables.

## Results

**Figure 1:**
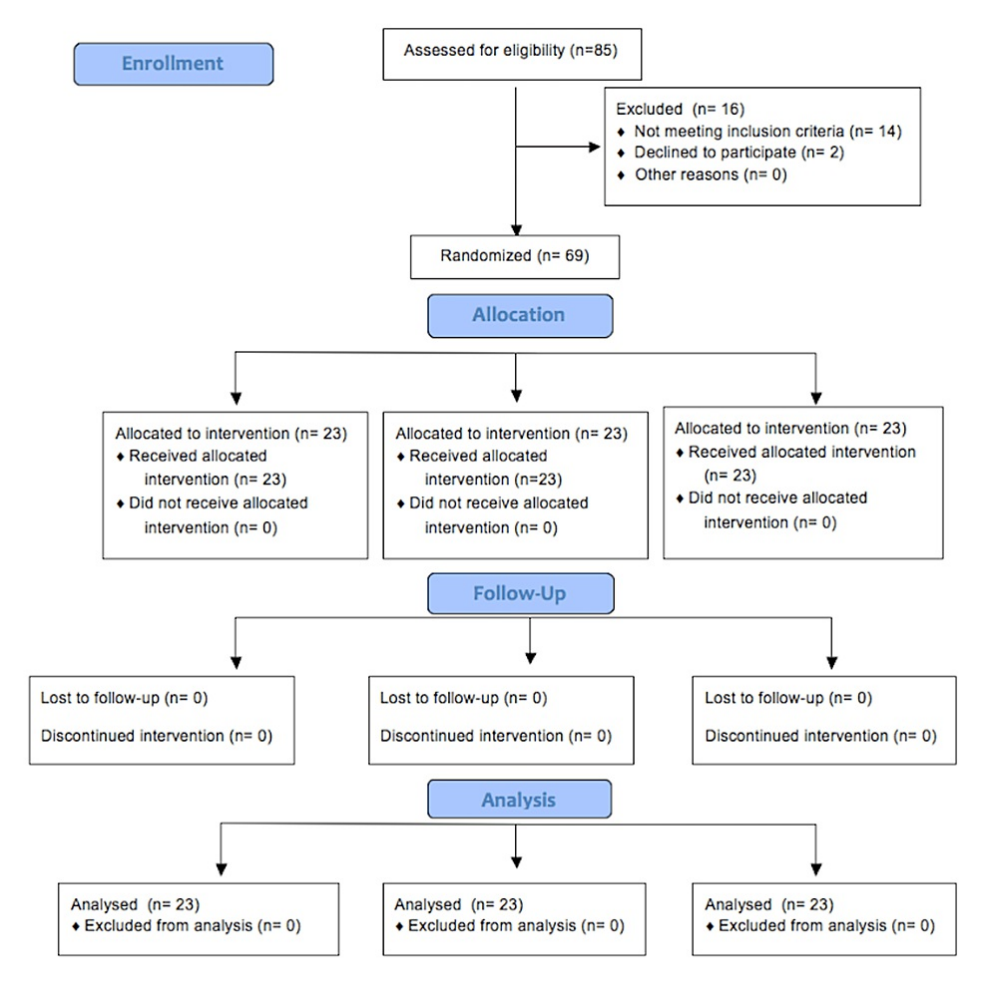
CONSORT flow diagram CONSORT: Consolidated Standards of Reporting Trials

Figure 1 shows the flow of patients through the trial in the Consolidated Standards of Reporting Trials (CONSORT) diagram.

Postoperatively, all the patients were followed up and observed for four hours in the post-anesthesia care unit as per our protocol. The three groups did not differ significantly with respect to ASA grade, age, and sex. Although the difference in weight and height between Group A and Group C came to be statistically significant, the difference of 2.22 cm in mean height and 5.39 kg in mean weight was clinically insignificant (Table 1).

**Table 1:** Demographics distribution

Parameter		Group A	Group B	Group C	P-value	Inter-group comparison (Tukey’s)
Age in years	Range	20-32	21-36	20-34	P=0.519	Non-significant
	Mean± SD	25.35±3.142	24.43±3.396	25.57±4.009		
Weight in kilograms	Range	58-78	56-78	58-86	P=0.007	Significant p-value between groups A and C
	Mean± SD	65.83±4.668	67.61±4.659	71.22±7.280		
Height in centimeters	Range	152-162	152-163	152-161	P=0.017	Significant p-value between groups A and C
	Mean± SD	157±1.859	156.22±2.999	154.78±2.746		

**Figure 2:**
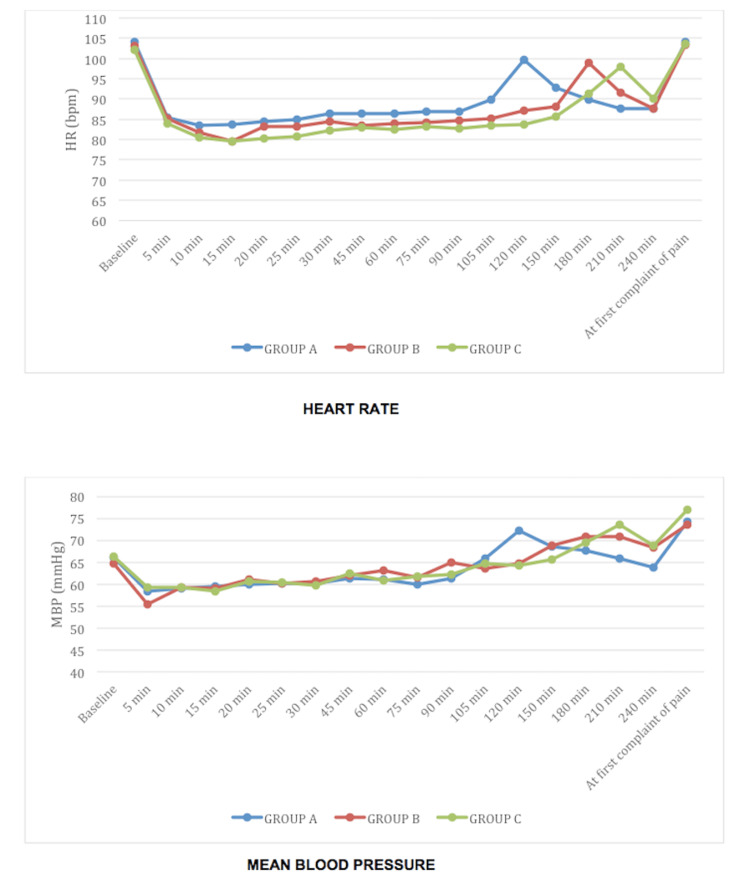
Hemodynamic parameters No significant changes were observed between the three groups HR: heart rate; MBP: mean blood pressure; Min: minutes

There was no significant variation in terms of hemodynamic parameters in the three groups (Figure 2).

Although the time of onset of sensory block was insignificant between the three groups, there was significant variation in time for two-segment regression of sensory block and duration of effective analgesia being more in nalbuphine groups (Group B and Group C) (Table 2).

**Table 2:** Characteristics of sensory block

Time in minutes	Group A (Mean ± SD)	Group B (Mean ± SD)	Group C (Mean ± SD)	p-value	Inter-group comparison (Tukey’s)
Time of onset of action	5.87±1.290	6.17±1.696	6.00±1.087	P=0.756	Not significant
Time of two-segment regression	65.00±7.071	85.87±15.348	101.74±8.996	p<0.001	p-value significant between groups A and B, A and C and B and C
Duration of effective analgesia	121.74±11.833	183.91±15.880	206.09±18.766	p<0.001	p-value significant between groups A and B, A and C and B and C

**Figure 3:**
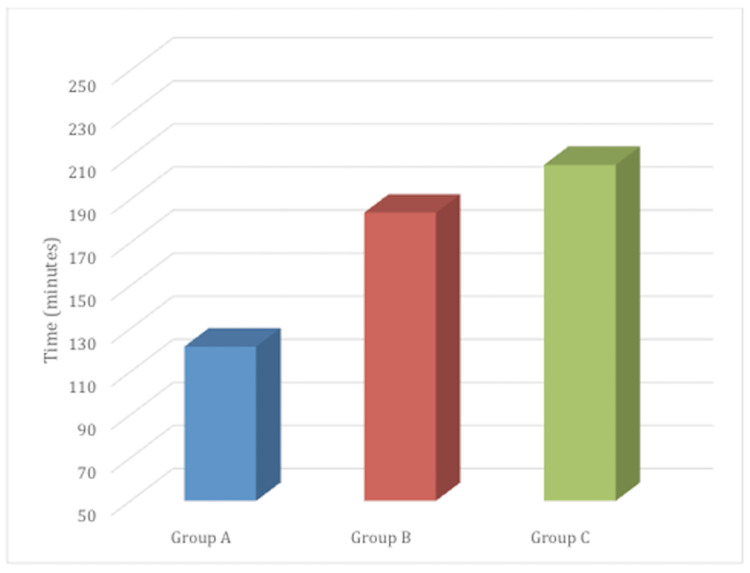
Duration of effective analgesia It was observed as the time between the subarachnoid block to first complaint of pain, having visual analogue score ≥ 3.

The duration of effective analgesia in our study was 121.74 ± 11.833 minutes in Group A, 183.91 ± 15.880 minutes in Group B, and 206.09 ± 18.766 minutes in Group C. The difference was statistically significant between all three pair of groups: A and B, A and C, and B and C (p<0.001) (Figure 3).

Different characteristics of motor blockade were studied in three groups (Table 3).

**Table 3:** Characteristics of motor blockade SAB: subarachnoid block

Parameters	Group A	Group B	Group C	P (Groups A and B)	P (Groups A and C)	P (Groups B and C)	P
Quality of motor block (good)	17 (73.913%)	22 (95.652%)	23 (100%)	0.048	0.011	0.50	P-value significant between groups A and B and A and C
Time from SAB to maximum level of motor block (minutes)	10.00±0.001	10.00±0.002	12.39±2.554	>0.05	<0.001	<0.001	P-value significant between groups Aand C and Band C
Time from SAB to complete regression of motor block (minutes)	174.78±14.73	203.48±20.138	216.52±15.553	<0.001	<0.001	0.03	P-value significant between all three groups

**Figure 4:**
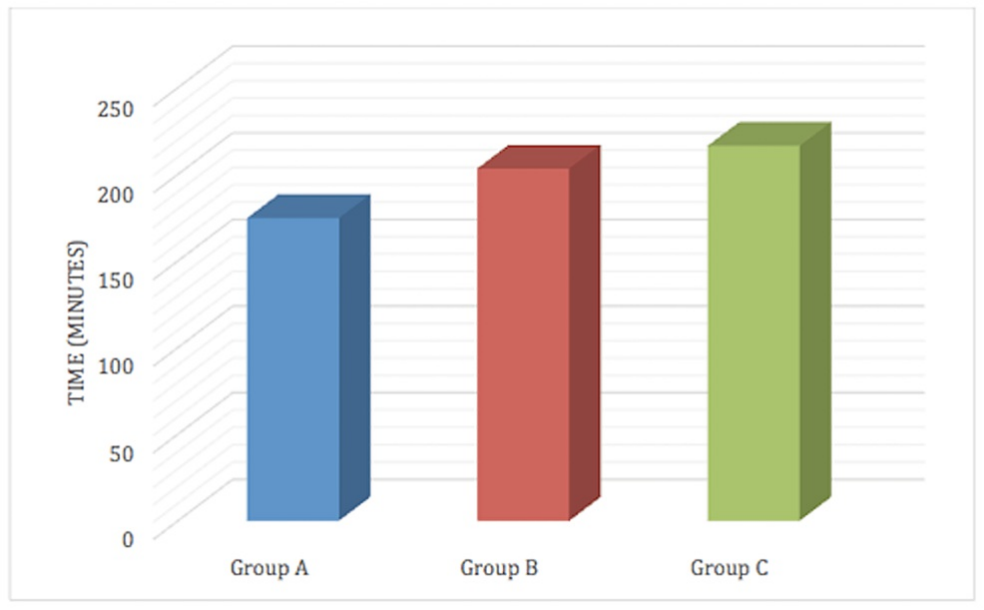
Time from SAB to complete regression of motor blockade SAB: subarachnoid block

The quality of block achieved in nalbuphine groups as assessed by the surgeon was significantly better than the plain ropivacaine group (Group A). The time for complete regression of motor blockade was significantly greater in nalbuphine groups (Group B and Group C), which further increased significantly with incremental dose (Figure 4).

The time of onset of SAB to baby delivery and completion of surgery were also comparable in the three groups. The block height is the maximum level of sensory block achieved after SAB. In Group A and Group B, the maximum sensory level ranged between T4-T6 with a median of T6. In Group C the maximum sensory level ranged between T4-T6 with a median of T4. Statistical analysis by the Mann-Whitney test showed that the difference in maximum block height was not significant between Groups A and Group B (p=0.16) while the difference was significant between groups A and C and groups B and C (P<0.001).

The F-test was chosen to determine the feature importance score of various parameters i.e. "Group (fixed dose ropivacaine + different dosage of nalbuphine/normal saline)", "Time from SAB to two-segment regression", "Time from SAB to motor block disappearance", "Maximum level of block height achieved", "height" and "weight" on the duration of effective analgesia. The feature importance score of "Group" was dominantly high compared to the other parameters. The height and weight parameters were less significant. Additionally, linear regression and decision trees were used to demonstrate that the exclusion of height and weight parameters did not have any significant effect on the performance of the models, further supporting the observation from the F-test.

Side effects were noted after the SAB in all three groups. The difference between the three groups was statistically not significant (Table 4).

**Table 4:** Side effects observed in the study groups

Side Effects	Group A	Group B	Group C
Nausea	3 (13.043%)	1 (4.347%)	4 (17.391%)
Vomiting	None	1 (4.347%)	1 (4.347%)
Hypotension	None	3 (13.043%)	3 (13.043%)
Bradycardia	None	1 (4.347%)	None
Pruritus	None	None	None
Other	None	None	None

Apgar score was observed in all newborns at one minute and five minutes. At all time periods, the Apgar score of the newborns was found to be ≥ 8. None of the newborns had an Apgar score of ≤ 7 at any time period in any group. The difference in the Apgar score of newborns between the three groups was not significant (p>0.05) (Table 5).

**Table 5:** Apgar score in three groups There was no significant difference observed between the three groups

Apgar Score	Group A		Group B		Group C	
	At 1 minute	At 5 minutes	At 1 minute	At 5 minutes	At 1 minute	At 5 minutes
10	2 (8.695%)	22 (95.652%)	7 (30.434%)	20 (86.956%)	3 (13.043%)	22 (95.652%)
9	21 (91.304%)	1 (4.347%)	16 (69.565%)	3 (13.043%)	19 (82.608%)	1 (4.347%)
8	0 (0%)	0 (0%)	0 (0%)	0 (0%)	1 (4.347%)	0 (0%)
≤7	0 (0%)	0 (0%)	0 (0%)	0 (0%)	0 (0%)	0 (0%)

The cord blood pH of all newborns was analyzed by Combisys II ABG machine (Eschweiler GmbH & Co. KG) within 10 minutes of collection of the sample. The difference in mean cord blood pH of newborns between the three groups was not significant (p=0.344).

## Discussion

Modern neuraxial techniques at childbirth reflect a shift in the practice of obstetric anesthesia, from a simple focus on pain relief towards a focus on overall quality of analgesia. Because of the provision of superior analgesia and materno-fetal benefits afforded by neuraxial techniques, its use has progressively increased in the recent past. In addition to this, the increasing knowledge of physiology and pharmacotherapy of pain and the development of obstetric anesthesia as a sub-specialty has led to an overall improvement in the quality of pain management during and after vaginal delivery and cesarean section [18-20]. Neuraxial techniques are preferred for cesarean delivery when compared to general anesthesia because of some major and obvious attributing factors like increasing use of epidural technique for labor analgesia, avoidance of risk of airway complications of general anesthesia in parturients, the ability of the mother to remain awake and experience childbirth, easy to perform, less expensive, reduction in stress response to surgery, early ambulation, and above all maternal satisfaction [21]. Our study compared two different doses of nalbuphine as adjuvant to isobaric ropivacaine in patients undergoing LSCS under SAB in terms of quality of sensory and motor block, hemodynamic parameters, duration of effective analgesia, Apgar score in newborns, and associated side effects. 

Ateseret al. evaluated the optimum dose of isobaric ropivacaine to be used intrathecally in pregnant women undergoing LSCS [5]. They randomly divided 60 pregnant women into three groups where groups 1, 2, and 3 received 15 mg, 20 mg, and 25 mg isobaric ropivacaine (1%) intrathecal, respectively. They did not find any significant difference in hemodynamic parameters between the three groups. Gomaa et al. compared intrathecal nalbuphine with bupivacaine and intrathecal fentanyl with bupivacaine, for postoperative analgesia after cesarean section [13]. They did not find any significant difference in hemodynamic parameters between the two groups. The result of our study in terms of hemodynamic parameters was comparable to that of the study conducted by Gomaa et al. and Mukherjee et al. [13,22].

For cesarean delivery, it has been proposed that to achieve optimum conditions for surgery, one should aim to achieve an upper level of sensory anesthesia of T4. In our study, the range of maximum spread of sensory block in all three groups was T4-T6 dermatome with a median of T6 in groups A and B, and T4 in Group C. Combining all three groups of our study, most participants achieved a maximum sensory block level of T6 dermatome; even after this, the surgery remained uninterrupted without any complaint of pain in any patient. These findings in our study were comparable to the studies conducted by Ateser et al., Bindra et al., and Gomaa et al. [5,9,13].

The dose and density of the anesthetic solution and the position of the patients are the most important factors affecting intrathecal drug spread. Compared with isobaric solutions, the use of hyperbaric local anesthetics results in a more predictable spread with less inter-patient variability [15]. Various studies have shown that the use of isobaric local anesthetic in SAB has been associated with greater variability in the spread of block and is less predictable, so that the block may either be too low, and therefore may be inadequate for surgery, or excessively high causing side effects [23,24]. However, in our study, the spread of isobaric ropivacaine alone or in combination with nalbuphine remained consistent, stable, and predictable, which can be explained by the fact that the density of cerebrospinal fluid is lower in premenopausal pregnant women making our study drug isobaric ropivacaine, a little hyperbaric compared to it [15].

The degree of loss of sensation assessed by the patients provides a subjective perspective of the quality of sensory block in terms of excellent, good, fair, or poor. In our study, more than 98% of patients combined in all three groups had an excellent degree of loss of sensation where there was a complete absence of sensation. Surgeons, based on muscle relaxation, assessed the quality of motor block as ‘good’, ‘fair’, or ‘poor’. Seventeen out of 23 patients in Group A, 22 out of 23 patients in Group B, and all 23 patients in Group C had ‘good’ motor blockade. Six patients in Group A and one (4.347%) patient in group B had a ‘fair’ muscle relaxation. The difference was statistically significant between groups A and B (p=0.048) and A and C (p=0.011) while the difference was statistically not significant between groups B and C (p=0.50).

The mean time of the two-segment regression of sensoryblock from the highest level achieved in our study was 65.00 ± 7.071 minutes in Group A, 85.87 ± 15.348 minutes in Group B, and 101.75 ± 8.996 minutes in Group C. The difference was statistically significant between all three pair of groups: A and B, A and C, and B and C (p<0.001). In the study conducted by Ateser et al., the time to two-segment regression of sensory block in the isobaric ropivacaine 15 mg group was 127.8 ± 43.22 minutes [5]. This difference in time to two-segment regression of sensory block from our study may be explained in part by the difference in height of patients between our and their patients and secondly by the use of higher doses of nalbuphine by them. The combination of intrathecal opioids with intrathecal local anesthetics limits the regression of sensory block seen with local anesthetics alone. Gomaa et al. used 0.8 mg nalbuphine with 2 ml of 0.5% heavy bupivacaine in LSCS under SAB and found that time to two-segment regression of sensory block was 123.00 ± 5.66 minutes [13]. In another study, Mukherjee et al. used nalbuphine with hyperbaric bupivacaine intrathecally in lower limb surgeries and found that time to two-segment regression of sensory block increased from 141.5 ± 5.83 minutes in nalbuphine 0.4 mg group to 153.3 ± 6.05 minutes in nalbuphine 0.8 mg group [22]. The time to two-segment regression of sensory block in the nalbuphine 0.6 mg group (Group C) in our study (101.75 ± 8.99 minutes) was comparable to that in the study conducted by Bindra et al. (108.46 ± 5.51 minutes) who used nalbuphine 0.8 mg along with bupivacaine in pregnant patients undergoing LSCS under SAB [9].

Duration of effective analgesia is an important parameter while assessing the quality of sensory block in SAB. In our study, the difference was statistically significant between all three pair of groups (p<0.001). The study drug provided postoperative analgesia of 50.65 minutes in Group A, 112.17 minutes in Group B, and 128.79 minutes in Group C. The study drug can explain the difference in the duration of effective analgesia among the three groups. Group A provided the least duration of effective analgesia while the same increased when nalbuphine was used as an adjuvant. Nalbuphine itself is an analgesic and adds to the effect of ropivacaine, thus prolonging the duration of effective analgesia. In the studies conducted by Culebras et al., Bindra et al., and Gomaa et al., the duration of effective analgesia in the parturient given nalbuphine 0.8 mg group was 212 ± 72 minutes, 259.20 ± 23.23 minutes, and 213.83 ± 15.73 minutes, respectively [6,9,13]. The longer duration of effective analgesia in their studies compared to our study can be attributed to the use of a higher dose (0.8 mg) of nalbuphine, higher cutoff VAS (≥ 4), and possibly due to the use of a different local anesthetic agent (bupivacaine heavy) by them.

The time from SAB to complete regression of motor block in our study was 174.78 ± 14.731 minutes in group A, 203.48 ± 20.138 minutes in group B, and 216.52 ± 15.553 minutes in group C. The difference was highly significant between the control group and the two groups with nalbuphine. Khaw et al. studied 72 patients undergoing elective LSCS in which the patients were randomized to receive different doses of spinal ropivacaine (10 mg, 15 mg, 20 mg, and 25mg) diluted to 3 ml volume with normal saline [17]. They found that the median of time from SAB to complete regression of motor block of ropivacaine 15 mg group was 120 minutes with a range of 90-240 minutes. The results of our study lie within this range. In another study conducted by Khaw et al. in 2002, they compared the characteristics of spinal anesthesia with plain and hyperbaric ropivacaine (25 mg) for elective cesarean delivery [25]. They found that the time from SAB to complete regression of the motor block of the 25 mg plain ropivacaine group was 219 minutes. The difference between the results of our study and the result of their study could be because of a higher dose of plain ropivacaine used by them.

Opioids when added to local anesthetics in SAB are associated with various side effects like hypotension, bradycardia, nausea, vomiting, pruritus, respiratory depression, etc. In our study, the incidence of nausea was 13.043% in Group A, 4.347% in Group B, and 17.391% in Group C, while the incidence of vomiting was 4.347% in groups B and C and none in Group A. The incidence of nausea and vomiting in our study was less in comparison to the results of Ateser et al., Culebras et al., and Gomaa et al. [5,6,13]. In cesarean section under SAB, nausea and vomiting can occur due to various factors, the most important being decreased cerebral blood flow as a consequence of hypotension. The reason for the lesser incidence of nausea and vomiting in our study may be due to a lower dose of local anesthetic used.

Another common side effect associated with SAB is hypotension. Epidural veins of pregnant females remain engorged because of aortocaval compression, as a result of which displacement of CSF occurs from the thoracolumbar area, which may cause unwanted cephalad extension of block increasing the risk of hypotension. The incidence of hypotension in our study combining all three groups was 8.69%. Only one patient in our study had an episode of bradycardia, which recovered after a single use of atropine. Pruritus, a common side effect is usually more common with the intrathecal use of hydrophilic opioids like morphine and less common with lipophilic opioids. In our study, none of the patients complained of pruritus. Nalbuphine has an antipruritic property, which is, in fact, used to treat the pruritus caused by the intrathecal use of other opioids. There were no other side effects found in any study group. In our study, the use of nalbuphine had no adverse effect on neonatal outcomes. The Apgar scores at one minute and five minutes showed no significant difference among the groups and the scores were within the physiological range in all three groups. None of the newborns had an Apgar score of ≤ 7 at any time period in any group. The findings in our study were comparable to the studies done by Culebras et al. and Gomaa et al. [6,13].

The mean cord blood pH of newborns in our study was 7.33±0.016 in Group A, 7.33±0.015 in Group B, and 7.32±0.016 in Group C. The difference was not significant (p=0.344). The cut-off value of cord blood pH for perinatal asphyxia was 7. In our study, perinatal asphyxia was not observed in any newborn. The finding in our study was comparable to the study done by Ateser et al. and Khaw et al. [5,25]. Due to their pharmacological profiles, ropivacaine and nalbuphine have no fetal toxicity. Furthermore for spinal anesthesia, only small quantities of drugs were used and the rate of drug absorption from the subarachnoid space was low, thereby reducing the potential fetal side effects.

Limitations of our study

Confounding factors like vertebral column length and abdominal girth for intrathecal drug spread were not observed.

## Conclusions

All three groups in our study provided adequate sensory and motor block for cesarean section, but the quality of motor blockade was better in adjuvant groups. The mean time to two-segment regression and the duration of effective analgesia was significantly longer with the use of 0.6 mg nalbuphine as an adjuvant to 2 ml of intrathecal 0.75 % isobaric ropivacaine. The difference in time from SAB to complete regression of motor block between the adjuvant groups (0.4 mg and 0.6 mg nalbuphine) was comparable. There were no clinically significant changes in hemodynamic and respiratory parameters in all three groups. Neonatal depression was not seen in any newborn. The difference in side effects among the three groups was not significant. We conclude that in comparison to 0.4 mg, 0.6 mg nalbuphine in combination with ropivacaine for SAB in patients undergoing LSCS provides a significantly longer duration of effective analgesia without any significant difference in side effects.
